# Construction of a Novel Prognostic Model in Lung Adenocarcinoma Based on 7-Methylguanosine-Related Gene Signatures

**DOI:** 10.3389/fonc.2022.876360

**Published:** 2022-06-16

**Authors:** Fei Lu, Jingyan Gao, Yu Hou, Ke Cao, Yaoxiong Xia, Zhengting Chen, Hui Yu, Li Chang, Wenhui Li

**Affiliations:** ^1^ Department of Radiation Oncology, The Third Affiliated Hospital of Kunming Medical University, Tumor Hospital of Yunnan Province, Kunming, China; ^2^ Department of Oncology and Hematology, Southern Central Hospital of Yunnan Province, The First People’s Hospital of Honghe State, Mengzi, China

**Keywords:** lung adenocarcinoma, 7-methylguanosine (m^7^G), RNA methylation, prognosis, tumor immune microenvironment

## Abstract

Increasing evidence has implicated the modification of 7-methylguanosine (m^7^G), a type of RNA modification, in tumor progression. However, no comprehensive analysis to date has summarized the predicted role of m^7^G-related gene signatures in lung adenocarcinoma (LUAD). Herein, we aimed to develop a novel prognostic model in LUAD based on m^7^G-related gene signatures. The LUAD transcriptome profiling data and corresponding clinical data were acquired from the Cancer Genome Atlas (TCGA) and two Gene Expression Omnibus datasets. After screening, we first obtained 29 m^7^G-related genes, most of which were upregulated in tumor tissues and negatively associated with overall survival (OS). According to the expression similarity of m^7^G-related genes, the combined samples from the TCGA-LUAD and GSE68465 datasets were further classified as two clusters that exhibit distinct OS rates and genetic heterogeneity. Then, we constructed a novel prognostic model involving four genes by using 130 differentially expressed genes among the two clusters. The combined samples were randomly divided into a training cohort and an internal validation cohort in a 1:1 ratio, and the GSE72094 dataset was used as an external validation cohort. The samples were divided into high- and low-risk groups. We demonstrated that a higher risk score was an independent negative prognostic factor and predicted poor OS. A nomogram was further constructed to better predict the survival of LUAD patients. Functional enrichment analyses indicated that cell cycle and DNA replication-related biological processes and pathways were enriched in the high-risk group. More importantly, the low-risk group had greater infiltration and enrichment of most immune cells, as well as higher ESTIMATE, immune, and stromal scores. In addition, the high-risk group had a lower TIDE score and higher expressions of most immune checkpoint-related genes. We finally noticed that patients in the high-risk group were more sensitive to chemotherapeutic agents commonly used in LUAD. In conclusion, we herein summarized for the first time the alterations and prognostic role of m^7^G-related genes in LUAD and then constructed a prognostic model based on m^7^G-related gene signatures that could accurately and stably predict survival and guide individualized treatment decision-making in LUAD patients.

## Introduction

Lung cancer, although experiencing a modest reduction in new cases globally, has remained the leading cause of cancer-related deaths for many years according to the latest epidemiologic statistics (1, 2). The 5-year survival rate of lung cancer increased from about 10% in 2000 to nearly 20% in 2014 despite great progress in its screening, diagnosis, and treatment (3). Based on histopathological classifications, lung adenocarcinoma (LUAD) is the most common subtype, affecting approximately 40% of lung cancer cases (4). Recent studies have suggested that LUAD is a highly heterogeneous disease at multiple levels, particularly at the molecular and gene levels (5). Therefore, a novel and more precise prognostic model based on genetic or epigenetic alterations is necessary to guide therapeutic decision-making and predict patient prognosis.

Increasing evidence has suggested that RNA modifications play a vital role among a variety of malignancies (6, 7). So far, >170 types of RNA modifications have been documented. Of these, RNA methylation, encompassing several types [N6-methyladenosine (m^6^A), 5-methylcytosine (m^5^C), N6-2′-O-dimethyladenosine (m^6^Am), N1-methyladenosine (m^1^A), and N7-methylguanosine (m^7^G)], is a major epigenetic modification (8). As one positively charged essential modification in messenger RNA (mRNA), m^7^G is installed at the 5′ cap co-transcriptionally during transcription initiation and can modulate nearly every phase of the mRNA life cycle and stabilize transcripts against exonucleolytic degradation (9–11). Besides functioning as a part of the cap structure, recent studies have further demonstrated the presence of internal mRNA m^7^G modifications, which could impact mRNA translation, and also confirmed methyltransferase-like 1 (*METTL1*) as a methyltransferase capable of installing a subset of m^7^G within mRNA (9, 12). In addition, m^7^G is one of the most common transfer RNA (tRNA) modifications when installed by *METTL1–WDR4* (WD repeat domain 4) at position 46 (m^7^G46) of tRNAs in humans (13). Concomitantly, m^7^G also occurs at position 1639 of 18S ribosomal RNA in mammals, having been installed by Williams–Beuren syndrome chromosome region 22 (WBSCR22) (14). These internal m^7^G modifications can impact RNA function and processing. Pandolfini et al. (15) recently showed that m^7^G methylation within microRNAs mediated by METTL1 could regulate cell migration. Taken together, these findings highlight the primary and critical role of m^7^G modification in the fates of mRNA, tRNA, ribosomal RNA, and microRNA in humans.

Recent studies have also implicated m^7^G in tumor progression and development in lung cancer, intrahepatic cholangiocarcinoma (ICC), hepatocellular carcinoma (HCC), bladder cancer, and colon cancer (16–21). Using bioinformatic analyses, accumulating studies have determined that the RNA modification-related gene signatures, such as m^6^A, m^1^A, and m^5^C, can predict the prognosis and guide therapeutic decisions in most cancers, including LUAD (22–27). However, to the best of our knowledge, there are no studies reporting the predictive role of m^7^G-related regulatory genes in any malignancies. In this study, by utilizing the expression data of m^7^G-related genes in LUAD from The Cancer Genome Atlas (TCGA) database and Gene Expression Omnibus (GEO) database, we comprehensively analyzed the genetic characteristics and prognostic value of m^7^G-related genes in LUAD, constructed a novel prognostic model based on m^7^G-related gene signatures, and further investigated the impacts on the tumor immune microenvironment (TIM), eventually evaluating drug sensitivity in different risk groups. Our findings contribute to a better understanding of the significant role of m^7^G-related gene signatures and provide novel insight for improving the clinical response to therapy in LUAD patients in the future.

## Materials and Methods

### Study Design

**Figure 1:**
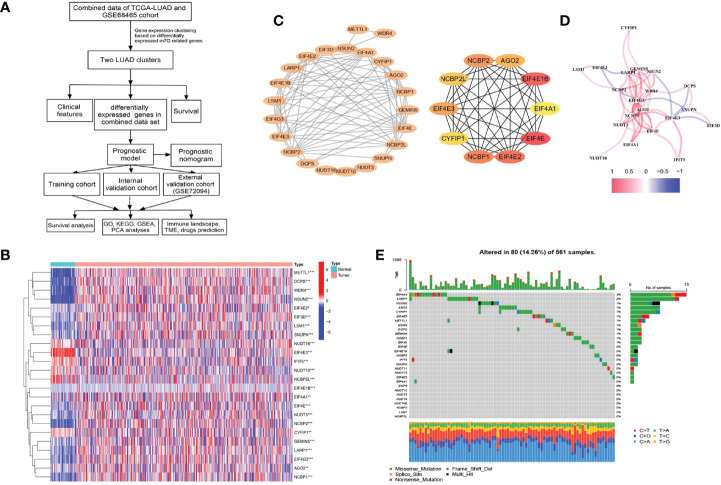
Characteristics and differences of m^7^G-related genes in The Cancer Genome Atlas-lung adenocarcinoma (TCGA-LUAD) cohort. **(A)** The flowchart of this study. **(B)** Heatmap for differences in m^7^G-related gene expression between LUAD tumor and normal tissues. **(C)** The PPI network between 24 differentially expressed m^7^G-related genes (*p* < 0.05) and the hub genes network. **(D)** The correlation in m^7^G-related gene expression. **(E)** Genetic mutation frequency and types of m^7^G-related genes. *p < 0.05, **p < 0.01, ***p < 0.001.

The flowchart of our study is depicted in **Figure 1A**.

### Data Acquisition and Preprocessing

The transcriptome profiling data and corresponding clinical data of LUAD were acquired from the TCGA database (https://portal.gdc.cancer.gov) and GEO database (https://www.ncbi.nlm.nih.gov/gds). In the TCGA cohort, the transcriptome data were downloaded as FPKM (fragments per kilobase per million mapped reads) and further converted to TPM (transcripts per million) using the “limma” R package for analysis. Then the “normalizeBetweenArrays” function of the R package “limma” was performed for data standardization. The TCGA-LUAD dataset including 535 tumor samples and 59 tumor-adjacent samples was used to compare the difference in the expression of m^7^G-related genes between tumor and normal tissues. For the GEO datasets, probe IDs were converted to gene symbols according to platform annotation files. Normalized expression values were log2-transformed and scaled before being used in model validations. Using the “combat” algorithm in the “sva” package of the R software, we correct the batch effect between the TCGA and GEO datasets. Genome mutation data of TCGA-LUAD [including somatic mutation and copy number variation (CNV)] were downloaded from the TCGA database and the UCSC Xena platform (https://gdc.xenahubs.net/). For the analyses involving clinical data, samples with unknown survival times were deleted.

### Identification of Differential Expression and Genetic Alterations in m^7^G-Related Genes

We identified m^7^G-related genes from published literature (28) and the gene sets named “m7G(5′)pppN diphosphatase activity”, “RNA 7-methylguanosine cap binding”, and “RNA cap binding” from the Molecular Signatures Database (MSigDB, https://www.gsea-msigdb.org/gsea/msigdb/search.jsp) with the keyword “7-methylguanosine.” To screen differentially expressed m^7^G-related genes (DEMGs) with the threshold of *p*-value <0.05 between tumor and normal adjacent tissues from the TCGA-LUAD dataset, the “limma” R package was used. After screening, the “heatmap” R package was applied for generating heatmaps. The immunohistochemistry (IHC) results from the Human Protein Atlas (HPA, https://www.proteinatlas.org) were used to validate the protein level of DEMGs in normal and tumor tissues. Meanwhile, we observed genetic mutation frequency and types of m^7^G-related genes in TCGA-LUAD samples by using the “maftools” R package.

### Construction of the Protein–Protein Interaction Network and Correlation Between m^7^G-Related Genes

Among proteins with co-expression coefficients >0.4, the STRING database (https://string-db.org/) was used to construct the protein–protein interaction (PPI) network. Cytoscape software (version 3.9.1) was used to visualize the network; moreover, the MCC algorithm of the cytoHubba plugin was used to screen the hub genes. The “reshape2” R package was used to identify the correlation between the expression of m^7^G-related genes.

### Identification of the Overall Survival-Associated m^7^G-Related Genes

Overall survival (OS) was assessed by the Kaplan–Meier method. The datasets of TCGA-LUAD and GSE68465 (29) were merged to explore the overall survival predictive value of each m^7^G-related gene. R packages “survival” and “survminer” were used.

### Hierarchical Clustering

To classify LUAD samples into different subgroups based on the m^7^G-related gene set, the R package “ConsensusClusterPlus” was used. We combined transcriptome profiling data and corresponding clinical data from the TCGA-LUAD and GSE68465 datasets for this analysis. The maximum number of clusters was nine. We selected 80% item resampling (pItem), 100% gene resampling (pFeature), a maximum evaluated *k* of 9, 50 resamplings (reps), kmeans (clusterAlg), Euclidean (distance), and a specific random seed (seed = 123456) in the R package “ConsensusClusterPlus” for this analysis. Based on the consensus matrices and the cumulative distribution function (CDF) curves of the consensus index, the optimum number of clusters was determined. The differences of survival and distribution of clinicopathologic characteristics were compared between different clusters and visualized using the R packages “survival”, “survminer”, “limma”, “ggplot2”, and “pheatmap”. “Gene set variation analysis (GSVA)” R packages were further used to explore the difference in biological processes between different clusters. We downloaded the gene set “c2.cp.kegg.v7.4.symbols” from the MSigDB database for this analysis. An adjusted *p*-value of less than 0.05 was considered statistically significant. A single-sample gene set enrichment analysis (ssGSEA) was used to quantify the enrichment scores and to represent the relative abundance of 23 tumor-infiltrating immune cell types between different clusters. The “maftools” R package was used to present mutational differences of each cluster. The CNV of different clusters was analyzed and visualized using the RCircos package in R.

### Construction and Validation of a Prognostic Model

Also, transcriptome profiling data and corresponding clinical data from the TCGA-LUAD and GSE68465 datasets were merged for this analysis. The combined data were randomly divided into a training cohort and an internal validation cohort in a 1:1 ratio. Meanwhile, the 442 LUAD samples from GSE72094 (30) were used as an external validation cohort. Briefly, by using the “limma” R package, the differentially expressed genes (DEGs) with adjusted *p*-value <0.05 and |log2FC| >0.585 between clusters were detected. Subsequently, univariate Cox analysis was applied to explore the prognosis-related DEGs. Then, we used the LASSO regression with 10-fold cross-validation to narrow down the prognosis-related DEGs applying the R package “glmnet,” and further performed the multivariate Cox regression analysis to establish a signature for evaluating the relationships between the DEGs and the survival of LUAD patients. The IHC results from the HPA were used to validate the protein level of this gene signature in normal and tumor tissues. Finally, we calculated the risk scores of each patient based on the following model formula: *risk score* = Σ*i Coefficient* (*mRNA_i_*) × *Expression*(*mRNA_i_*). According to the median risk scores in the training group, patients were separated into high- and low-risk groups among both training and validation cohorts. The Kaplan–Meier method was performed to compare the OS between the high- and low-risk groups. The predictive value of the prognostic model was assessed through time-related receiver operating characteristic (ROC). The heatmaps were used to compare and visualize the distribution of clinicopathologic characteristics between the risk cohorts. Multivariate Cox regression analysis was applied to test the prognostic independence of risk score. The principal component analysis (PCA) and t-distributed stochastic neighbor embedding (t-SNE) which can get a low-dimensional cluster distribution from high-dimensional gene sets were utilized for validating the classification results. The risk scores of each patient were further combined with clinical characteristics to construct a nomogram through the “rms” R package.

### Comparison of the Novel Prognostic Model and Previously Reported Models using ROC Curves and Concordance Index Values

To compare our prognostic model with other models previously reported in LUAD, four studies primarily focusing on m^6^A-related signatures were selected (22, 31–33). We extracted the genes included in these prognostic models and used ROC curves and concordance index (C-index) values to compare the predictive accuracy between different models. The R packages “survcomp,” “survival,” “ggplot2,” “ggpubr,” “limma,” “survminer,” and “timeROC” were applied.

### Functional and Pathway Enrichment Analyses

To explore the potential mechanisms and pathways between the high- and low-risk groups, the Gene Ontology (GO), Kyoto Encyclopedia of Genes and Genomes (KEGG) functional enrichment analysis, and gene set enrichment analysis (GSEA) were conducted among DEGs between the high- and low-risk groups using the R packages “clusterProfiler,” “enrichplot,” “limma,” “ggplot2,” and “org.Hs.eg.db.”

### Analyses of Immune Cells, Immune-Related Functions, and the Tumor Microenvironment

Using the ssGSEA through the “GSVA” R package, we compared the enrichment scores, represented the relative abundance of 23 tumor-infiltrating immune cell types between the high- and low-risk groups, and then visualized the results through the R packages “limma,” “ggpubr,” and”reshape2.” The differential analyses of stromal score, immune score, ESTIMATE score, and immune cells were performed based on the results of CIBERSORT and ESTIMATE using the R software packages “CIBERSORT” and “estimate.” Moreover, the mRNA expression-based stemness index (mRNAsi) scores of LUAD were obtained from previous research (34). Spearman’s correlation analyses were used to establish the relationship between risk scores and immune cells as well as between risk scores and mRNAsi scores. The tumor immune dysfunction and exclusion (TIDE) score (35) was calculated online (http://tide.dfci.harvard.edu/) to assess the immune checkpoint inhibitor (ICI) response between the high- and low-risk groups. We also compared the expressions of 47 immune checkpoint-related genes (**Supplementary Table 1**) between the high- and low-risk groups through the R software packages “limma,” “ggpubr,” and “ggplot2.”

### Chemotherapeutic and Small Molecule Drug Screening and Prediction

The public dataset, Genomics of Drug Sensitivity in Cancer (GDSC, https://www.cancerrxgene.org) (36), was chosen to evaluate the response to chemotherapeutic and small molecule drugs between the high- and low-risk groups, which was done by computing the half-maximum inhibitory concentration (IC_50_). The analyses were conducted through the “pRRophetic” R package.

### Statistical Analyses

All analyses were completed by using R language (Version 4.1.2). Student’s *t*-test, chi-squared test, or Wilcoxon test was applied to compare the differences between groups. Spearman’s correlation test was performed to evaluate the association between variables. A *p*-value of <0.05 was considered statistically significant.

## Results

### Screening and Genetic Landscape of m^7^G-Related Genes

**Figure 2:**
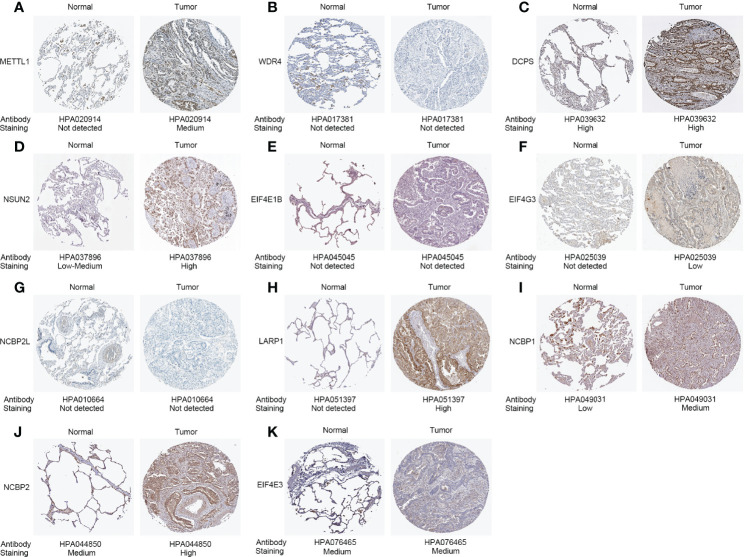
Protein expressions of 11 differentially expressed m^7^G-related genes in the tumor and normal tissues from the Human Protein Atlas platform. **(A)** METTL1 expression. **(B)** WDR4 expression. **(C)** DCPS expression. **(D)** NSUN2 expression. **(E)** EIF4E1B expression. **(F)** EIF4G3 expression. **(G)** NCBP2L expression. **(H)** LARP1 expression. **(I)** NCBP1 expression. **(J)** NCBP2 expression. **(K)** EIF4E3 expression.

After screening, we found 29 m^7^G-related genes, namely, AGO2, CYFIP1, DCP2, DCPS, EIF3D, EIF4A1, EIF4E, EIF4E1B, EIF4E2, EIF4E3, EIF4G3, GEMIN5, IFIT5, LARP1, LSM1, METTL1, NCBP1, NCBP2, NCBP2L, NCBP3, NSUN2, NUDT10, NUDT11, NUDT16, NUDT3, NUDT4, NUDT4B, SNUPN, and WDR4. Of these, 12 DEMGs with the threshold of |log2FC| >0.585 and p <0.05 were observed between 535 tumor and 59 normal TCGA-LUAD tissues, which consisted of 2 downregulated (NCBP2L, EIF4E3) and 10 upregulated (DCPS, EIF4E1B, EIF4G3, LARP1, LSM1, METTL1, NCBP1, NCBP2, NSUN2, and WDR4) DEMGs in tumor samples (**Figure 1B** and **Table 1**). The protein expressions of DEMGs in normal and tumor tissues were further validated using the IHC results from the HPA platform. As shown in **Figure 2**, the protein expressions of METTL1, NSUN2, EIF4G3, LARP1, NCBP1, and NCBP2 were higher in tumor tissues than in normal tissues; however, the protein expressions of WDR4, EIF4E1B, and NCBP2L were negative in both tumor and normal tissues. The PPI network between 24 DEMGs with the threshold of p <0.05 is shown in **Figure 1C**; of these DEMGs, EIF4E, EIF4E1B, EIF4E2, NCBP1, NCBP2, EIF4E3, AGO2, NCBP2L, CYFIP1, and EIF4A1 were the top 10 hub genes. Spearman’s correlation analysis suggested that EIF4E3 was most frequently and negatively correlated with other DEMGs, as shown in **Figure 1D**. A total of 561 samples in the TCGA-LUAD cohort were used for somatic mutation of m^7^G-related genes; among them, 80 (14.26%) samples were observed to have experienced mutation events. A missense mutation was the most common type of variant classification, and EIF4G3, LARP1, NSUN2, AGO2, and CYFIP1 were the top 5 most frequently mutated genes (**Figure 1E**).

**Table 1:** Differences in the expression of m^7^G-related genes in the TCGA-LUAD cohort.

Gene	Normal tissue	Tumor tissue	Log2FC	*p*
METTL1	3.53626	10.15226	1.521504	9.60E−30
WDR4	2.104496	4.390468	1.0609	2.74E−25
NSUN2	8.98576	20.20226	1.168804	1.40E−29
DCPS	3.496315	6.175647	0.820755	1.79E−24
NUDT10	0.17005	0.199114	0.227639	0.000217
NUDT16	9.429715	8.582916	−0.13575	0.000331
NUDT3	2.28048	3.011322	0.401059	1.81E−09
AGO2	3.348328	4.644651	0.472129	0.002944
CYFIP1	15.10077	14.09259	−0.09968	0.001197
EIF4E	2.014382	2.535048	0.331676	9.25E−06
EIF4E1B	0.001917	0.188505	6.619734	0.000329
EIF4E2	9.342995	10.33663	0.14581	0.026165
EIF4E3	5.466021	2.852976	−0.93802	4.09E−26
GEMIN5	4.074254	5.318471	0.384475	2.69E−07
LARP1	17.49505	27.5081	0.65291	5.73E−17
NCBP1	5.233116	7.854471	0.585844	1.28E−15
NCBP2	11.49588	17.48301	0.604837	1.21E−20
EIF3D	37.79962	47.5054	0.32972	5.80E−09
EIF4A1	1.539405	2.132041	0.469863	0.001368
EIF4G3	6.026233	9.566647	0.666757	1.91E−16
IFIT5	10.44892	8.593445	−0.28205	4.65E−08
LSM1	8.176932	12.76115	0.642126	1.82E−12
NCBP2L	0.154783	0.060228	−1.36175	6.79E−06
SNUPN	3.814629	4.839386	0.343281	2.41E−07

m^7^G, 7-methylguanosine; TCGA-LUAD, The Cancer Genome Atlas-lung adenocarcinoma; FC, fold change.

### Survival Analysis Based on the Expression of Each m^7^G-Related Gene

**Figure 3:**
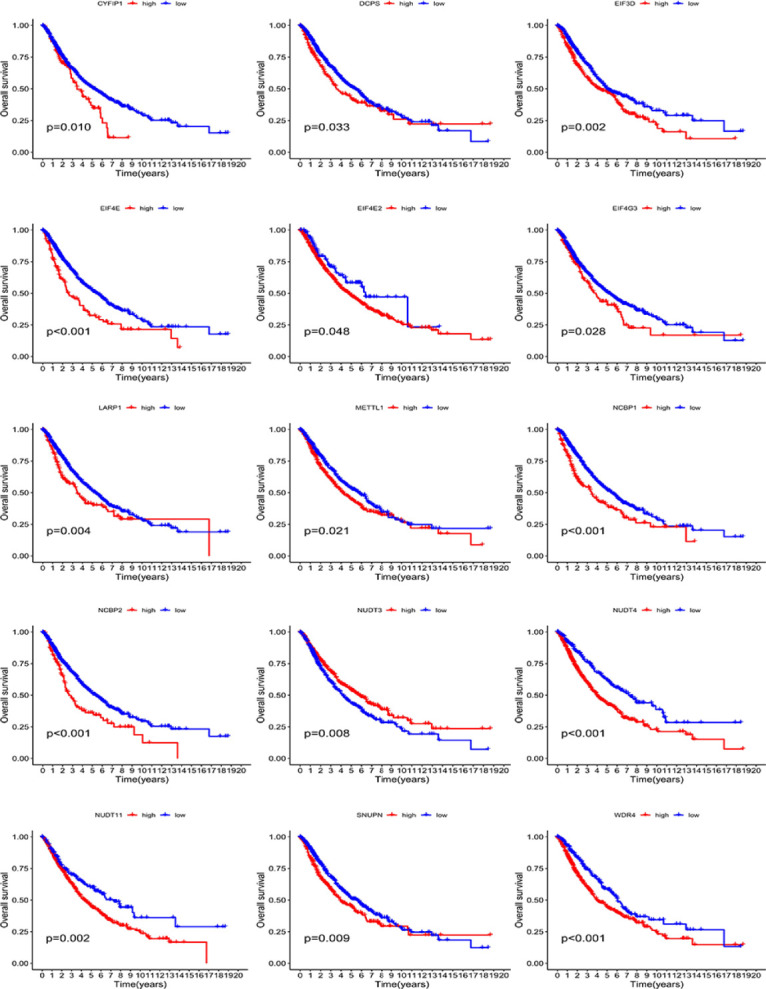
The overall survival analysis based on the expression of each m^7^G-related gene.

To further verify the predictive role of each m^7^G-related gene, we conducted survival analyses using the Kaplan–Meier method. As shown in **Figure 3**, the high expression of most genes (including *CYFIP1*, *DCPS*, *EIF3D*, *EIF4E*, *EIF4E2*, *EIF4G3*, *LARP1*, *METTL1*, *NCBP1*, *NCBP2*, *NUDT4*, *NUDT11*, *SNUPN*, and *WDR4*) portended a significantly poor OS. Conversely, a high expression of *NUDT3* predicted an improved OS (**Figure 3**).

### Consensus Clustering Based on the Expression of m^7^G-Related Genes

**Figure 4:**
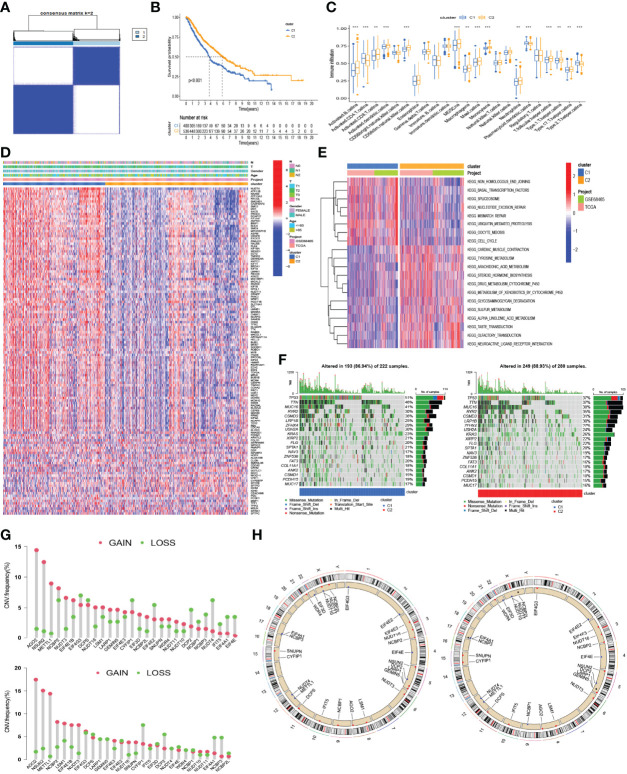
Consensus clustering based on the expression of m^7^G-related genes. **(A)** The heatmap of the consensus matrix showing that 2 was the appropriate *k* value. **(B)** Kaplan–Meier curves for the OS in patients with different clusters. **(C)** The differences in the scores of immune cells between the two clusters. **(D)** Heatmap for the distribution of clinicopathologic characteristics and the difference of the expression of 130 DEGs between the two clusters. **(E)** Heatmap for the difference of biological process in GSVA enrichment analysis based on the KEGG gene set. **(F)** The waterfall plot showing the differences in somatic genomic mutation between cluster 1 (C1) and cluster 2 (C2). **(G)** Histogram reflecting the copy number variation (CNV) of the m^7^G-related genes in C1 (up) and C2 (down). **(H)** The location of CNV alteration of m^7^G-related genes on 23 chromosomes in C1 (left) and C2 (right). **p < 0.01, ***p < 0.001.

Based on the expression similarity of the 29 m^7^G-related genes, the consensus clustering method was applied to cluster the combined LUAD samples of the TCGA and GSE68465 cohorts. The cluster number *k* ranged from 2 to 9; when *k* = 2, a relatively clear-cut boundary was shown in the heatmap of the consensus matrix (**Figure 4A**, **Supplementary Figure S1A**), and a flat slope was seen in the CDF curve of the consensus index score (**Supplementary Figure S1A**). Hence, we selected *k* = 2 as the appropriate number of clusters and divided 936 LUAD samples into two clusters—namely, cluster 1 (C1, *n* = 400) and cluster 2 (C2, *n* = 536). Next, Kaplan–Meier survival analysis was applied to evaluate the prognostic value of this clustering. A significant difference in OS was observed between the two clusters (log-rank *p* < 0.001). C1 had a worse median OS (**Figure 4B**). Accordingly, the samples of C1 had higher levels of gene expression compared with C2, as shown in the heatmap (**Figure 4D**). Also, as seen in the heatmap, however, variations in other clinicopathological characteristics did not show statistical significance between the samples of each cluster. Similarly, C1 had a higher expression of most m^7^G-related genes compared with C2 (**Supplementary Figure S1B**). The results of the GSVA enrichment analysis based on the KEGG gene set showed that the process of cell cycle and DNA repair, such as non-homologous end-joining, spliceosome, nucleotide excision repair, mismatch repair, and cell cycle, was enriched in C1; C2 was prominently enriched in metabolism-associated pathways, such as tyrosine metabolism, arachidonic acid metabolism, drug metabolism cytochrome P450, metabolism of xenobiotics by cytochrome P450, sulfur metabolism, and alpha-linolenic acid metabolism (**Figure 4E**). From the results of the ssGSEA, we found that the scores of some immune cells, such as activated B cells, activated CD8 T cells, activated dendritic cells (aDCs), CD56 dim nature killer cells, myeloid-derived suppressor cells (MDSCs), macrophages, mast cells, monocytes, neutrophils, T follicular helper cells, type 1 T helper cells, and type 17 T helper cells, were significantly enriched in C2 (**Figure 4C**). In addition, the somatic mutations were more frequent in C1 than in C2 (**Figure 4F**). The frequencies and locations of the CNVs were also different in C1 and C2 (**Figures 4G, H** **)**, and the copy number losses were more frequent in C2 than in C1.

### Construction and Validation of a Novel Prognostic Model Based on DEGs Between Clusters

**Figure 5:**
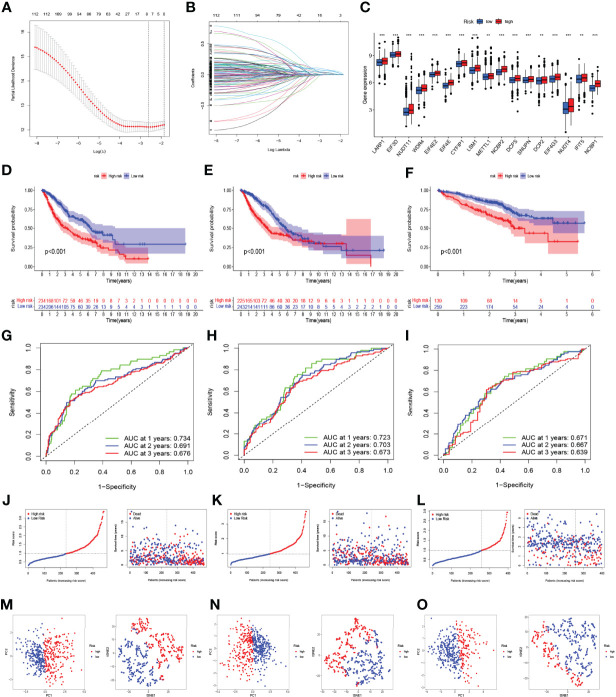
Construction and validation of the prognostic model based on the m^7^G-related gene signatures in lung adenocarcinoma (LUAD). **(A, B)** LASSO analysis with minimal lambda value. **(C)** The difference in the expression of m^7^G-related genes in the high- and low-risk groups. The Kaplan–Meier survival analysis showing the difference in overall survival (OS) between the high- and low-risk groups in the training **(D)**, internal validation **(E)**, and external validation cohorts **(F)**. Time-dependent ROC curve analysis in the training **(G)**, internal validation **(H)**, and external validation cohorts **(I)**. The distribution of risk score and survival status of LUAD patients with different risk scores in the training **(J)**, internal validation **(K)**, and external validation cohorts **(L)**. PCA and t-SNE analyses in the training **(M)**, internal validation **(N)**, and external validation cohorts **(O)**. **p < 0.01, ***p < 0.001.

Using the “limma” R package, the DEGs were first screened between the two clusters in a combined LUAD dataset of the TCGA and GSE68465 cohorts, and 130 DEGs were finally obtained. Then, we used the univariate Cox analysis to explore 112 prognosis-related DEGs. To prevent model overfitting, LASSO penalized Cox regression modeling was conducted to screen the key DEGs associated with survival. With this method, a novel prognostic gene model with four genes was constructed (**Figures 5A, B** **)**. Subsequently, risk scores per sample were calculated using the following model formula: risk score = (0.1606739599952 × expression value of *KIF20B*) + (0.207218473949824 × expression value of *HMMR*) + (0.157719134596455 × expression value of *ARNTL2*) + (0.0802509860697548 × expression value of *DKK1*). The combined LUAD dataset was randomly divided into a training cohort and internal validation cohort in a 1:1 ratio, and the 442 LUAD samples from the GSE72094 dataset were used as an external validation cohort. The samples were divided into high-risk and low-risk groups according to the median threshold of risk scores in the training group (**Figures 5J–L**). The expressions of most m^7^G-related genes were significantly higher in the high-risk group than in the low-risk group (**Figure 5C**). As shown by the Kaplan–Meier analyses, patients in the high-risk group had significantly worse OS than those in the low-risk group (*p* < 0.001, **Figures 5D–F**); analogously, as the risk score increased, more patients died (**Figures 5J–L**). In the training cohort, the AUC values of the present risk model were 0.734, 0.691, and 0.676 for the 1-, 2-, and 3-year prognoses, respectively (**Figure 5G**), with similar results observed in the internal and external validation groups (**Figures 5H, I** **)**. The distribution patterns from t-SNE and PCA analyses showed that samples could completely be distinguished into high- and low-risk groups (**Figures 5M–O**). Taken together, these findings demonstrated the prognostic robustness of the novel prognostic model in patients with LUAD.

### Comparison of Our Prognostic Model and Previously Reported Models Using ROC Curves and C-Index Values

**Figure 6:**
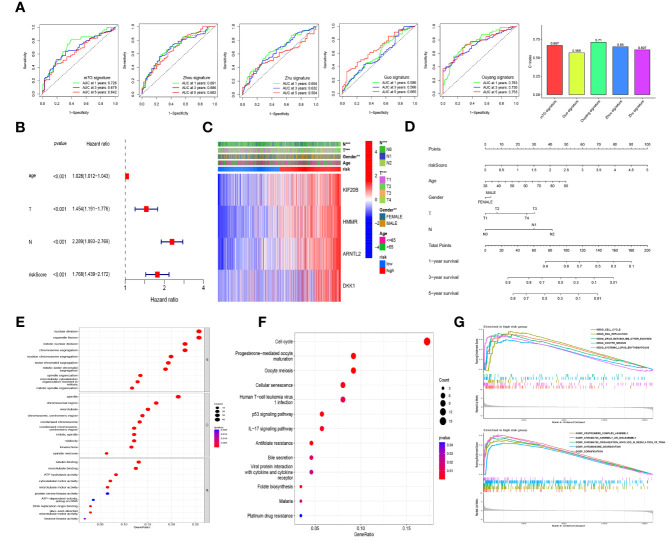
Model comparison, independent prognostic factor analysis, clinical correlation analysis, nomogram construction, and functional and pathway enrichment analyses in the different risk cohorts. **(A)** The comparison of our prognostic model and previously reported models using ROC curves and concordance index (C-index) values. **(B)** The multivariate Cox regression analysis of the risk score and other clinical features in the training cohort. **(C)** Heatmap for the distribution of clinicopathologic characteristics between the high- and low-risk groups in the combined lung adenocarcinoma (LUAD) dataset of the TCGA and GSE68465 cohorts. **(D)** A nomogram using risk scores combined with clinical characteristics. GO enrichment analysis **(E)** and KEGG pathway analysis **(F)** based on the differentially expressed genes between the high- and low-risk groups in the combined LUAD dataset. **(G)** Gene set enrichment analysis (GSEA) based on KEGG and GO in the high-risk group in the combined LUAD dataset. ** *p*<0.01, ***p< 0.001.

To compare our prognostic model with other models previously reported in LUAD, we searched four studies primarily focusing on m^6^A-related signatures (22, 31–33). The number of genes included in these models varied from 3 to 27. We found that the AUC and C-index values of our model were higher than those of other models except for the model constructed by Ouyang et al. (32) (**Figure 6A**).

### Independent Prognostic Factor Analysis, Clinical Correlation Analysis, Nomogram Construction, and Functional and Pathway Enrichment Analyses

Univariate and multivariate Cox regression analyses were performed by introducing age, gender, TNM stage, and risk scores to assess the independence of risk scores in the survival prediction of LUAD patients. Those variables with *p <*0.1 in the univariate analysis were selected for multivariate analysis. Among the samples in the training cohort, the results showed that age, T stage, N stage, and risk score were identified as independent negative prognostic factors for patients with LUAD (**Figure 6B**), and similar results were observed in the internal and external validation groups (**Supplementary Figures S2A, B**). Meanwhile, in the internal validation cohort, risk score was of greater assistance than the other clinical characteristics in predicting prognosis (HR = 1.689, 95% CI: 1.376–2.075, *p* < 0.001; **Supplementary Figure S2A**). As shown in **Figure 6C**, the heatmap presented the distribution of clinicopathological features between the high- and low-risk cohorts. There were significant differences in N stage (*p* < 0.01), T stage (*p* < 0.001), and gender (*p* < 0.01) between the different risk groups. To facilitate the utilization of our risk model, we further constructed a nomogram using risk scores combined with clinical characteristics, as shown in **Figure 6D**, and calibration curves further verified this nomogram as reliable and accurate in predicting 1-, 3-, and 5-year OS (**Supplementary Figure S2G**).

By considering the DEGs between the high- and low-risk groups from combined samples of the TCGA and GSE68465 cohorts, we conducted GO enrichment analysis, KEGG pathway analysis, and GSEA to explore the potential biological functions of these genes. As shown in **Figure 6E** and **Supplementary Figure S2D**, “nuclear division” and “organelle fission” were the most enriched terms among the biological process categories, and “spindle” and “tubulin binding” were the most enriched terms among the cellular component and molecular function categories, respectively. Cell cycle was identified to be the most enriched among the KEGG pathways of the DEGs (**Figure 6F** and **Supplementary Figure S2C**). GSEA was further performed to identify the differential pathways enriched in GO and KEGG between the high- and low-risk subgroups, and the results revealed that cell cycle and DNA replication-related biological processes and pathways were enriched in the high-risk group; however, the biological processes and pathways enriched in the low-risk group were weakly associated with tumor initiation and progression (**Figure 6G** and **Supplementary Figure S2E**).

### Risk Signature-Based Immune Cell Infiltration, Immune-Related Pathways, Tumor Microenvironment, and Stemness Analyses

**Figure 7:**
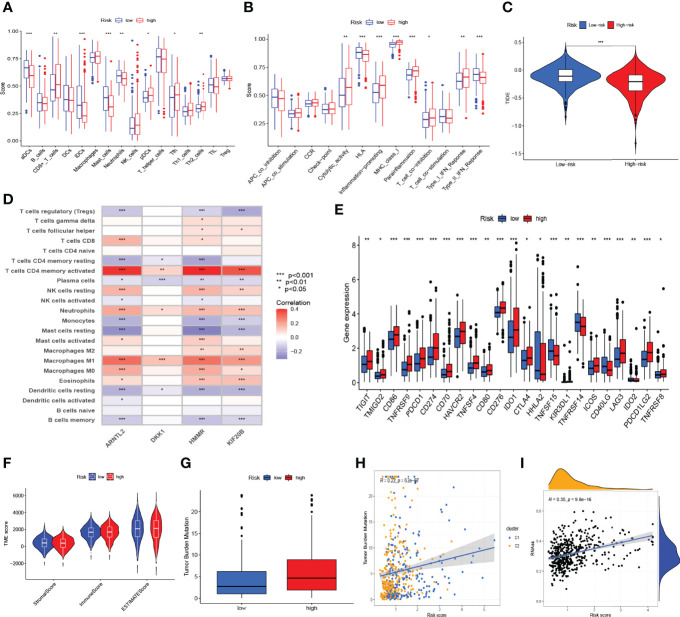
Risk signature-based immune cell infiltration, immune-related pathways, tumor microenvironment (TME), and stemness analyses. The differences in the scores of immune cells **(A)** and immune functions **(B)** in the training cohort. **(C)** The differences in tumor immune dysfunction and exclusion (TIDE) score in The Cancer Genome Atlas-lung adenocarcinoma cohort. **(D)** The correlations of immune cell infiltration and the four genes in the risk model in a combined lung adenocarcinoma (LUAD) dataset of the TCGA and GSE68465 cohorts. **(E)** The differentially expressed immune checkpoint-related genes between the high- and low-risk groups. **(F)** ESTIMATE, immune, and stromal scores between the high- and low-risk groups in the combined LUAD dataset. **(G)** The difference in tumor mutation burden (TMB) between the high- and low-risk groups in the combined LUAD dataset. Spearman’s correlation analyses between the risk score and TMB **(H)**, as well as between the risk score and mRNAsi scores (RNAss) **(I)** in the combined LUAD dataset. *p < 0.05, **p < 0.01, ***p < 0.001.

ssGSEA was performed to quantify the enrichment scores of 13 immune cell-related functions and 16 immune cells between the two risk groups. Intriguingly, the scores of some immune cells, such as aDCs, immature dendritic cells (iDCs), mast cells, and neutrophils, were significantly enriched in the low-risk group of the training cohort (**Figure 7A**). However, the scores of some immune functions, such as cytolytic activity, inflammation promoting, major histocompatibility complex (MHC) class I, parainflammation, type-I interferon (IFN) response, and T-cell co-inhibition, were significantly enriched in the high-risk group (**Figure 7B**). Similar results were observed in the internal testing dataset (**Supplementary Figure S2F**). We further analyzed the correlations between immune cell infiltration and the expression of genes involved in the construction of the prognostic model, as shown in **Figure 7D**. The high-risk group had a lower TIDE score (**Figure 7C**) and higher expressions of most immune checkpoint-related genes (**Figure 7E**), which suggested that patients in the high-risk group may benefit from immunotherapy. In addition, we observed that patients in the low-risk group had higher ESTIMATE, immune, and stromal scores (**Figure 7F**). Additionally, by using Spearman’s correlation analysis, a positive and significant correlation was observed between risk score and tumor mutation burden (TMB, *R* = 0.22, *p* < 0.001, **Figures 7G, H** **)** as well as between risk score and mRNAsi score (RNAss, *R* = 0.35, *p* < 0.001, **Figure 7I**), which demonstrated that LUAD patients with higher risk scores had higher RNAss values and TMBs.

### Drug Screening Based on the m^7^G-Related Risk Signature

We further evaluated the response to chemotherapeutic and small molecule drugs between the high- and low-risk groups, as described previously. In the high-risk group, a total of 57 drugs with obviously lower IC_50_ values were observed; concomitantly, a total of 28 drugs were observed in the low-risk group (**Table 2**). Based on the drugs commonly used to treat LUAD, we noticed that patients in the high-risk group were more sensitive to chemotherapeutic agents such as cisplatin, docetaxel, etoposide, gemcitabine, paclitaxel, and vinorelbine.

**Table 2:** The sensitive chemotherapeutic and small molecule drugs in the high- and low-risk groups.

Group	Sensitive drugs
High risk	A-443654, ABT-263, ABT-888, AG-014699, AICAR, ATRA, AUY922, AZD7762, BAY 61-3606, BI-2536, BI-D1870, BIBW2992, bleomycin, BMS-708163, bortezomib, bosutinib, BX-795, camptothecin, CCT018159, CGP60474, CGP-082996, cisplatin, CMK, cytarabine, docetaxel, doxorubicin, epothilone B, etoposide, gemcitabine, GW843682X, JNK inhibitor VIII, JW.7.52.1, KU-55933, midostaurin, mitomycin C, NU-7441, NVP-TAE 684, obatoclax mesylate, paclitaxel, parthenolide, pazopanib, pyrimethamine, QS11, rapamycin, RO-3306, S-trityl-L-cysteine, SL 0101-1, thapsigargin, TW-37, vinblastine, vinorelbine, vorinostat, VX-680, X17-AAG, X681640, Z-LLNle-CHO, ZM-447439
Low risk	AMG-706, AS601245, AZ628, AZD6244, bexarotene, bicalutamide, bryostatin 1, CCT007093, DMOG, EHT-1864, erlotinib, FH535, GDC0941, GNF-2, GW 441756, imatinib, JNK 9L, lapatinib, LFM-A13, metformin, MK-2206, nutlin-3a, PAC-1, PD-0332991, roscovitine, salubrinal, VX-702, WO2009093972

## Discussion

With the growing in-depth understanding of RNA modifications, m^7^G modifications have gradually become a research hotspot in recent years. However, there is still a lack of comprehensive analyses to summarize the role of m^7^G-related gene signatures in LUAD. In this study, we comprehensively analyzed the genetic characteristics and prognostic value of m^7^G-related genes in LUAD and then further constructed a novel prognostic model based on m^7^G-related gene signatures to predict survival and guide treatment decisions.

We first extensively screened the m^7^G-related genes and finally obtained 29 genes, namely, AGO2, CYFIP1, DCP2, DCPS, EIF3D, EIF4A1, EIF4E, EIF4E1B, EIF4E2, EIF4E3, EIF4G3, GEMIN5, IFIT5, LARP1, LSM1, METTL1, NCBP1, NCBP2, NCBP2L, NCBP3, NSUN2, NUDT10, NUDT11, NUDT16, NUDT3, NUDT4, NUDT4B, SNUPN, and WDR4. Most genes have been reported to modulate some phases of the RNA life cycle, especially in mRNA (28, 37–41). In the TCGA-LUAD dataset, we found that DCPS, EIF4E1B, EIF4G3, LARP1, LSM1, METTL1, NCBP1, NCBP2, NSUN2, and WDR4 were significantly upregulated in tumor tissues than in adjacent normal tissues (|log2FC| > 0.585, p < 0.05), which were further validated in protein levels using the IHC results from the HPA platform; moreover, high expressions of DCPS, EIF4G3, LARP1, METTL1, NCBP1, NCBP2, and WDR4 predicted a significantly poor OS. METTL1, as one of the key tRNA-modifying enzymes, has been extensively reported to promote cancer development by mediating tRNA m^7^G modifications in lung cancer, ICC, HCC, and bladder cancer (16–20). Consistent with our results, Ma et al. (16) found that METTL1 and WDR4 were upregulated in lung cancer, and METTL1 promoted lung cancer growth and invasion via regulation of m^7^G tRNA modification *in-vitro* and *in-vivo* assays. Also, METTL1 inhibition can improve the sensitivity of HeLa cells to 5-fluorouracil (42). These results suggested that METTL1 may be a target for LUAD therapy. In a study reported by Huang et al. (43), EIF4G3, encoding a eukaryotic translation initiation factor involved in mRNA cap recognition and transport of mRNAs to the ribosome, was found to be a direct target of miR-375 in lung squamous cell carcinoma cells, and silencing of EIF4G3 induced cell apoptosis and suppressed tumor growth. LARP1 is an RNA-binding protein that regulates the 5′-terminal oligopyrimidine tract mRNA (44). Recent studies have revealed that LARP1 drives oncogenesis, and higher levels of LARP1 protein correspond with a poor prognosis in NSCLC, colorectal cancer, prostate cancer, ovarian cancer, HCC, and ICC (45–51). Xu et al. (45) found that LARP1 knockdown inhibited cell proliferation, migration, invasion, and tumorigenic potential in NSCLC cells, which can be regulated by the XIST/miR-374a axis. In another study, LARP1 was established as a target of miR-503 and further regulated by circ-BANP to promote lung cancer progression. LARP1 can also regulate mTOR signaling to contribute to cancer progression (52). These observations, combined with ours, suggest that LARP1 should be acknowledged as an oncogene and could be a promising therapeutic target in LUAD. NCBP1, which can participate in transcriptional and post‐transcriptional processes together with NCBP2 and NCBP3, mediated the proliferation, migration, and invasion of LUAD cells through upregulation of CUL4B (53). Interestingly, we noted that NUDT3, as a Nudix protein possessing mRNA-decapping activity in cells, was upregulated in tumor tissues of LUAD (|log2FC| = 0.4 and p < 0.001), but its high expression predicted significantly better survival. Grudzien-Nogalska et al. (54) reported that a reduction in NUDT3 protein levels in MCF-7 cells promoted cell migration. Unfortunately, there is no evidence to suggest the underlying role of NUDT3 in LUAD, which requires further investigations.

According to the expression similarity of the 29 m^7^G-related genes, the combined LUAD samples were further classified into two clusters, and C1 had a worse median OS than C2. Consistent with the survival analysis, C1 had a higher level of gene expression than C2. Concomitantly, the biological process from the KEGG gene set, the scores of some immune cells, the somatic mutations, and the frequencies and locations of the CNVs between the two clusters were different. However, differences in clinicopathological characteristics, such as T stage, N stage, gender, and age, did not have statistical significance between the clusters. These findings suggest that the prognostic variations of different clusters might mainly be due to the genetic heterogeneity of LUAD patients. Therefore, we used 130 DEGs between the two clusters to construct a novel prognostic model with four genes (*KIF20B*, *HMMR*, *ARNTL2*, and *DKK1*). Of these DEGs, KIF20B (also known as MPHOSPH1), a kinesin protein that plays a critical role in cytokinesis, has been found to promote the progression of some cancers such as clear cell renal cell carcinoma (55), pancreatic cancer (56), hepatocellular carcinoma (57, 58), tongue cancer (59), and bladder cancer (60) by stimulating cell proliferation. HMMR (hyaluronan-mediated motility receptor), also called RHAMM/CD168, has been extensively reported to promote the progression of LUAD and can serve as a key prognostic biomarker for patients with LUAD (61–64). Moreover, Brady et al. (65) have found a positive association between the expression of the transcription factor ARNTL2 (aryl hydrocarbon receptor nuclear translocator-like 2) and the outcome of patients with LUAD. ARNTL2 is a paralog of the circadian transcription factor ARNTL and has recently been discovered to also act as a modifier of immune cell infiltration in malignancies (66–68). Meanwhile, the Wnt antagonist DKK1 (Dickkopf-1) has been implicated in the modulation of immune cell activities as well as the immunosuppressive microenvironment in cancers and has become a promising target for cancer immunotherapy (69, 70). Together, these findings confirmed the reliability and precision of our prognostic model. Subsequently, the combined LUAD dataset of the TCGA and GSE68465 cohorts was randomly divided into a training cohort and internal validation cohort in a 1:1 ratio, and the dataset from GSE72094 was further used as an external validation cohort. The samples were divided into high-risk and low-risk groups according to the median threshold of the risk score in the training group. By using survival, time-dependent ROC, PCA, t-SNE, and univariate and multivariate Cox regression analyses, we noticed that a higher risk score was a negative predictive factor for survival and was identified as one of the independent negative prognostic factors for patients with LUAD. These findings further demonstrated the prognostic robustness of the novel prognostic model in patients with LUAD. Some clinicopathological characteristics, such as age, T stage, and N stage, were also identified as independent negative prognostic factors for patients with LUAD. Therefore, we further constructed a nomogram using risk scores combined with clinical characteristics to better predict the survival of LUAD patients.

We also compared our prognostic model with other four models previously reported in LUAD in studies primarily focused on m^6^A-related signatures. We found that the AUC and C-index values of our models were higher than those of the other models, except for the signature by Ouyang et al. (32). The study reported by Ouyang et al. (32) constructed a novel prognostic model including 27 genes for LUAD based on hypoxia, immunity, and epithelial–mesenchymal transition gene signatures; the overall survival differed significantly between the high-risk and low-risk groups (HR = 4.26), and the AUC values for predicting 1-, 3-, and 5-year survival were 0.763, 0.766, and 0.728, respectively. Despite a better precision, this model was not conducive to clinical translation due to the inclusion of too many genes.

We then conducted GO enrichment analysis, KEGG pathway analysis, and GSEA, considering DEGs between the high- and low-risk groups. The results implied that cell cycle and DNA replication-related biological processes and pathways may contribute to LUAD progression regulation by m^7^G-related gene signatures. We also found that most immune cells, ESTIMATE scores, immune scores, and stromal scores were enriched in the low-risk group, which suggested that m^7^G-related gene signatures may affect LUAD survival outcomes by altering the TIM and tumor microenvironment (TME). However, the high-risk group had a lower TIDE score and higher expressions of most immune checkpoint-related genes, which suggested that patients in the high-risk group may benefit from immunotherapy. We finally screened chemotherapeutic and small molecule drugs that were sensitive to different risk groups. We noticed that patients in the high-risk group were more sensitive to commonly used chemotherapeutic agents in LUAD, such as cisplatin, docetaxel, etoposide, gemcitabine, paclitaxel, and vinorelbine. This was presumably due to the enrichment of cell cycle and DNA replication-related biological processes and pathways in the high-risk group, and these drugs may interfere with the cell cycle *via* different mechanisms.

To the best of our knowledge, this is the first bioinformatics analysis to elucidate the prognostic roles of m^7^G gene signatures in malignancies. However, some limitations should be considered in the interpretation of our results. First, our study is a retrospective study based on three public datasets, and further large-scale and prospective studies are needed for validation. Second, the biological process of m^7^G modification has not been illustrated as thoroughly as that of m^6^A modification until now, so the m^7^G-related genes in our study may not be able to fully summarize all the processes of m^7^G modification. Third, further investigations will be required to determine the role of m^7^G modifications in LUAD development and progression.

In conclusion, we have summarized for the first time the alterations and prognostic role of m^7^G-related regulatory genes in LUAD and then constructed a prognostic model based on m^7^G-related gene signatures involving four genes, which can accurately and stably predict survival and guide individualized treatment decisions in LUAD patients. We further found that alterations in immune cell infiltration and TME characteristics may be a potential mechanism of this model to predict the prognosis of LUAD patients.

## Data Availability Statement

The original contributions presented in the study are included in the article/**Supplementary Material**. Further inquiries can be directed to the corresponding authors.

## Author Contributions

Conception and design: FL, JG, and WL. Acquisition and interpretation of data: FL, YH, KC, and HY. Analysis of data: FL, LC, YX, and ZC. Visualization of results: KC, ZC, and JG. Initial manuscript writing: FL, JG, and KC. Revision of the manuscript: WL, LC, and YH. All authors read and approved the final manuscript.

## Funding

This study was funded by the National Natural Science Foundation of China (Nos. 81860536 and 82060558), Yunnan Fundamental Research Projects (Nos. 202001AS70011, 202001AY070001-075, and 202101AY070001-162), Ten-Thousand Talents Program of Yunnan Province (Yunling scholar, Youth talent), Yunnan Provincial Training Funds for Middle-Young Academic and Technical Leader Candidate (No. 202005AC160025), Yunnan Provincial Training Special Funds for High-Level Health Technical Personnel (Nos. L-2018001 and D-2019030), and Graduate Student Innovation Foundation of Kunming Medical University (Grant No. 2021D22).

## Conflict of Interest

The authors declare that the research was conducted in the absence of any commercial or financial relationships that could be construed as a potential conflict of interest.

## Publisher’s Note

All claims expressed in this article are solely those of the authors and do not necessarily represent those of their affiliated organizations, or those of the publisher, the editors and the reviewers. Any product that may be evaluated in this article, or claim that may be made by its manufacturer, is not guaranteed or endorsed by the publisher.
