# Supplementary material for: Construction of a Novel Prognostic Model in Lung Adenocarcinoma Based on 7-Methylguanosine-Related Gene Signatures
Source: Front Oncol. 2022 Jun 16;12:876360. doi: 10.3389/fonc.2022.876360 (PMC9243265; doi:10.3389/fonc.2022.876360)
Supplement: Supplementary file 1 [file DataSheet_1.docx]

Supplementary Material

## Supplementary Figures


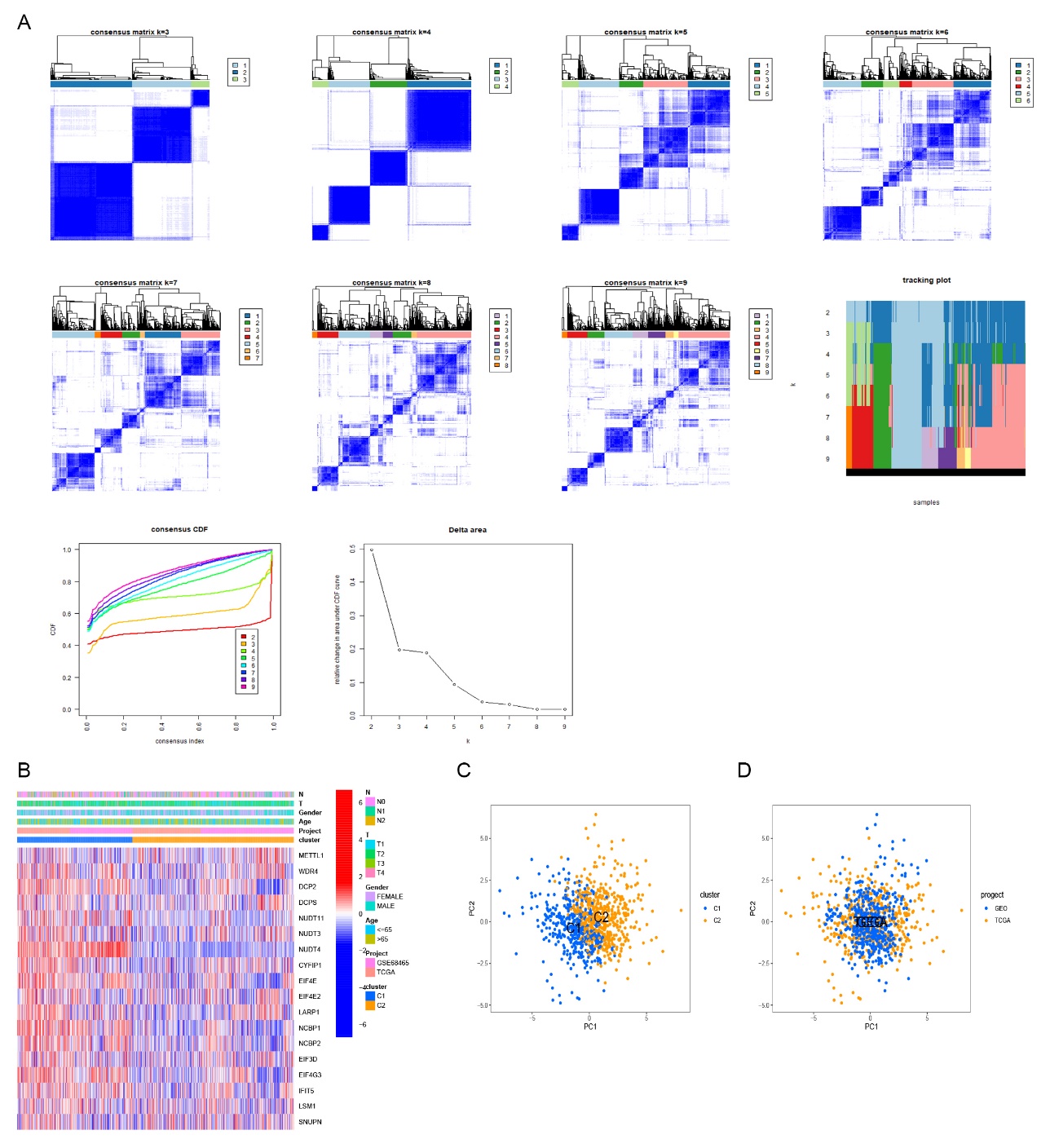


**Supplementary Figure S1.** Consensus clustering based on the expression of m^7^G-related genes. (A) The heatmap of consensus matrix, tracking plot, and the CDF curve showing that 2 was the appropriate K value. (B) Heatmap for the distribution of clinicopathologic characteristics and the difference of m^7^G-related genes expression between the 2 clusters. (C) PCA analysis in the 2 clusters. (D) PCA analysis for the distribution of TCGA and GEO in the batch-corrected combined samples.

**
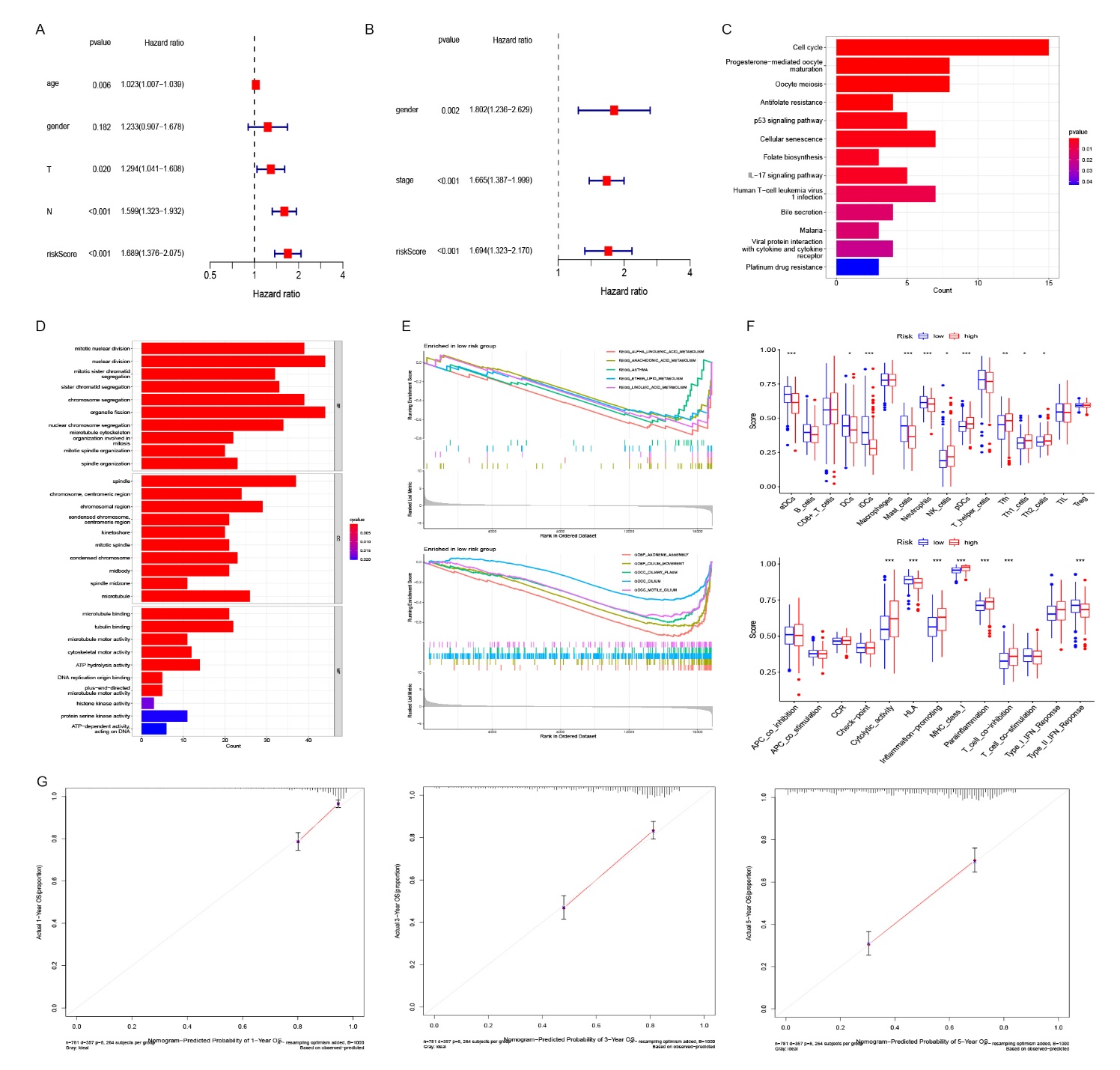
**

**Supplementary Figure S2.** Independent prognostic factor analysis, functional and pathway enrichment analyses in the different risk cohorts, risk signature-based immune cell infiltration, immune-related pathways, and nomogram construction. The multivariate Cox regression analysis of the risk score and other clinical features in the internal (A) and external validation cohort (B). KEGG pathway analysis (C) and GO enrichment analysis (D) based on the differentially expressed genes between the high- and low-risk groups in a combined lung adenocarcinoma (LUAD) dataset of the TCGA and GSE68465 cohorts. (E) Gene set enrichment analysis (GSEA) of based on KEGG and GO in the low-risk group among the combined LUAD dataset. (F) The differences of the scores of immune cells and immune functions in the internal testing dataset (G) The calibration curve of nomogram for the prediction and observed 1-, 3-, and 5-year overall survival (OS). **p* < 0.05, ***p* < 0.01, ****p* < 0.001.

**1.2 Supplementary Table**

**Supplementary TABLE 1** The overview of selected 47 immune checkpoints–related genes.

|  | Immune checkpoints–related genes |
| --- | --- |
| Gene Symbol | IDO1, LAG3, CTLA4, TNFRSF9, ICOS, CD80, PDCD1LG2, TIGIT, CD70, TNFSF9, ICOSLG, KIR3DL1, CD86, PDCD1, LAIR1, TNFRSF8, TNFSF15, TNFRSF14, IDO2, CD276, CD40, TNFRSF4, TNFSF14, HHLA2, CD244, CD274, HAVCR2, CD27, BTLA, LGALS9, TMIGD2, CD28, CD48, TNFRSF25, CD40LG, ADORA2A, VTCN1, CD160, CD44, TNFSF18, TNFRSF18, BTNL2, C10orf54, CD200R1, TNFSF4, CD200, NRP1 |
